# The effect of tacrolimus-induced toxicity on metabolic profiling in target tissues of mice

**DOI:** 10.1186/s40360-022-00626-x

**Published:** 2022-11-28

**Authors:** Dadi Xie, Jinxiu Guo, Ruili Dang, Yanan Li, Qingying Si, Wenxiu Han, Shan Wang, Ning Wei, Junjun Meng, Linlin Wu

**Affiliations:** 1 Tengzhou Central People’s Hospital, Tengzhou, 277500 China; 2 Translational Pharmaceutical Laboratory, Jining First People’s Hospital, Jining, 272000 China; 3 Department of Gastroenterology, Shanting District People’s Hospital, Zaozhuang, 277200 China

**Keywords:** Calcineurin inhibitor, Drug toxicity, Gas chromatography−mass spectrometry, Metabolome, Main tissues

## Abstract

Tacrolimus (Tac) is a common immunosuppressant that used in organ transplantation. However, its therapeutic index is narrow, and it is prone to adverse side effects, along with an increased risk of toxicity, namely, cardio-, nephro-, hepato-, and neurotoxicity. Prior metabolomic investigations involving Tac-driven toxicity primarily focused on changes in individual organs. However, extensive research on multiple matrices is uncommon. Hence, in this research, the authors systemically evaluated Tac-mediated toxicity in major organs, namely, serum, brain, heart, liver, lung, kidney, and intestines, using gas chromatography−mass spectrometry (GC-MS). The authors also employed multivariate analyses, including orthogonal projections to the latent structure (OPLS) and t-test, to screen 8 serum metabolites, namely, D-proline, glycerol, D-fructose, D-glucitol, sulfurous acid, 1-monopalmitin (MG (16:0/0:0/0:0)), glycerol monostearate (MG (0:0/18:0/0:0)), and cholesterol. Metabolic changes within the brain involved alterations in the levels of butanamide, tartronic acid, aminomalonic acid, scyllo-inositol, dihydromorphine, myo-inositol, and 11-octadecenoic acid. Within the heart, the acetone and D-fructose metabolites were altered. In the liver, D-glucitol, L-sorbose, palmitic acid, myo-inositol, and uridine were altered. In the lung, L-lactic acid, L-5-oxoproline, L-threonine, phosphoric acid, phosphorylethanolamine, D-allose, and cholesterol were altered. Lastly, in the kidney, L-valine and D-glucose were altered. Our findings will provide a systematic evaluation of the metabolic alterations in target organs within a Tac-driven toxicity mouse model.

## Introduction

Tacrolimus (Tac), a calcineurin inhibitor, is a highly efficacious immunosuppressive medication given during kidney, heart, lung, intestinal and bone marrow transplantation [1]. However, its efficient window is quite narrow, and it is challenging to sustain target Tac concentration in the blood. In clinical settings, even with Tac concentrations within therapeutic range, some patients experience rejection or toxicity. Hence, anorexia and organ toxicity are common occurrences. The major adverse reactions from Tac include nephrotoxicity, neurotoxicity, diabetogenesis, gastrointestinal disturbances, and hypertension [2, 3]. These adverse effects are usually dose-dependent. Unfortunately, despite multiple investigations on Tac and numerous hypotheses on its meschanisms, including Tac’s metabolite-related toxicity [3], accelerated apoptosis [4, 5], and augmented inflammation [6], the true mechanism behind Tac-mediated toxicity remains undetermined.

Metabolomics is a robust analytical strategy that identifies global complexity and important alterations in metabolites [7]. This technique provides overall metabolic profiling parameters in biological systems, and it is optimal for metabolic investigations. Our prior metabolomic investigations, using gas chromatography−mass spectrometry (GC-MS), identified numerous metabolites, which revealed alterations in metabolism under pathophysiological conditions [8]. Hence, it is essential to examine Tac-mediated toxicity. In prior investigations, Tac toxicity was examined by analyzing metabolic changes in target tissues like the serum, urine, heart, lung, and kidney [9–12]. Nevertheless, a systematic evaluation of Tac-driven toxicity in several biological systems is urgently needed. This is crucial for the determination of pathogenesis and Tac-mediated toxicity axis. Herein, our aim was to elucidate the metabolic outcomes of Tac-mediated toxicity on the mouse serum, brain, heart, liver, lung, kidney, and intestines. To achieve this, the authors performed a GC − MS, as well as uni- and multivariate analyses, to identify metabolic indicators in major murine tissues.

## Materials and methods

### Animal model

Our animal protocols strictly followed the Guide for Care and Use of Laboratory Animals (Chinese Council) and ARRIVE guidelines, and they received ethical approval from the Jining first people’s hospital (protocol number: JNRM2021DW105). Male KM (Kunming) mice, 6 weeks old, from the Jining medical college were conditioned to the animal facility environment for one week. When they reached 8 weeks of age, mice were arbitrarily separated into two groups: Tac and control (*n* = 9 per group). The Tac mice were then intraperitoneally administered with Tac (5 mg/kg, 0.1 mL/10 g of body weight) once a day for 14 days. The control mice received the same amount of normal saline instead. Our Tac dosage was selected, based on our prior research [13, 14]. Mice weight was recorded every day.

### Reagents

Tac was purchased from MedChemExpress (Shanghai, China). Heptadecanoic acid (purity: ≥98%; lot: SLBX4162), an internal standard (IS), and N,O-bis- (trimethylsilyl) trifluoroacetamide with 1% trimethylchlorosilane (BSTFA + 1% TMCS; v/v; lot: BCBZ4865) were acquired from Sigma-Aldrich (Saint Louis, MO, USA). o-Methyl hydroxylamine hydrochloride (purity: 98.0%; lot: LG10T16) was obtained from J&K Scientific Ltd. (Beijing, China). Pyridine (lot: C10551455) was purchased from Shanghai Macklin Biochemical (Shanghai, China). Chromatographic-grade methanol was acquired from Thermo Fisher Scientific (Waltham, MA, USA), and water from Hangzhou Wahaha Company (Hangzhou, China).

### Tissue sampling

Following treatments, animals were euthanized by cervical dislocation and blood and organs were extracted immediately. Total blood was collected from the eyes and underwent centrifugation at 5000 rmp for 6 min to retrieve the serum. The brain, heart, liver, lung, kidney, and intestines were harvested, PBS-rinsed, and instantly frozen in liquid nitrogen, then stored in − 80 °C until further analysis.

### Sample preparation

One hundred μL serum was combined with 350 μL methanol (with 100 μg/mL IS), and centrifuged (14,000 rpm, 4 °C, 10 min). The resulting supernatant was placed in 2 mL tubes, prior to drying at 37 °C using nitrogen gas. Next, the extracts were combined with 80 μL of o-methyl hydroxylamine hydrochloride (15 mg/mL in pyridine), prior to incubation at 70 °C for 90 min. Then, 100 μL of BSTFA + 1% TMCS was introduced to each sample, and incubated at 70 °C for 60 min. The mixture next underwent vortex, centrifugation at 14,000 rpm for 2 min at 4 °C, and filtering (0.22 μm) prior to GC − MS analysis.

Fifty mg of tissue (brain, heart, liver, lung, kidney, and intestines) underwent homogenization with 1 mL methanol and 50 μL 1 mg/mL IS, prior to transfer to a 2 mL tube, followed by centrifugation at 14,000 rpm, 4 °C for 10 min. The remaining procedure was the same as for serum samples.

### Serum and GC-MS analysis

Blood samples were collected and measure the serum concentration of FBG (fasting blood glucose), Crea (creatinine), Urea and ALT (alanine aminotransferase) by automatic biochemical analyzer.

Sample (serum, brain, heart, liver, lung, kidney and intestines) quality control (QC) represented a combination of samples from Tac and control mice. The retention time (RT) stability was assessed via the RT of IS. GC-MS analysis was performed on a 7890B GC system, attached to a 7000C MS. Sample separation was done using a HP-5MS fused-silica capillary column. Individual 1 μL aliquot of our prepared solution was run in split mode (50:1), with a helium flow rate of 1 mL/min. The GC temperature program was initiated at 60 °C for 4 min, then raised by 8 °C/min to 300 °C, and it was maintained at 300 °C for 5 min. The injection, transfer line, and ion source temperatures were 280 °C, 250 °C, and 230 °C, respectively. Electron impact ionization (− 70 eV) was employed with an acquisition rate of 20 spectra/s in the MS setting. The MS results were documented across the mass range of 50–800 m/z, using electrospray ionization.

### Multivariate statistical analyses

All quantitative data are expressed as the means ± SD. Statistical analysis was conducted via two-tailed Student’s t-test (SPSS 19.0, Chicago, USA); using a *p*-value threshold of < 0.05. GC data preprocessing was done via MassHunter Unknowns Analysis and Quantitative Analysis (Agilent Technologies). SIMCA-P v14.0 (Umetrics, Umea, Sweden) was employed for data analysis. Orthogonal projection to latent structures discriminant analysis (OPLS-DA) was performed to delineate between Tac and control groups, with a variable importance in projection (VIP) value > 1.0 and a *p*-value < 0.05 as the significance threshold. The model validity was confirmed via permutation evaluations (200 permutations). MetaboAnalyst v5.0 (http://www.metaboanalyst.ca) and Kyoto Encyclopedia of Genes and Genomes (KEGG; http://www.kegg.jp) were employed for functional analyses, and a raw *p*-value < 0.05 and impact > 0 were set as the significance threshold, as previously described [15].

## Results

### Biochemical alterations between the two groups

In this study, as shown in Table 1. The mean body weight at the beginning of the study was 27.87 ± 2.25 g. At time of sacrifice, the control mice had a mean body weight of 34.97 ± 5.63 g, and Tac mice 34.78 ± 2.96 g. There was no significant difference in weight between the control group and the groups treated with Tac (*P* > 0.05). The FBG, Crea, and urea were significantly higher in the group treated with Tac, compared to the control group (*P* ≤ 0.01). Furthermore, the ALT of the Tac group was increased more obviously compared with the control group (*P* ≤ 0.05).

**Table 1:** Weight and Serum FBG, Crea, Urea, ALT levels of control and Tac mice

Group	Weight(g)	FBG (mmol/L)	Crea (mg/dl)	Urea (mg/dl)	ALT(U/L)
Control	34.97 ± 5.63	5.09 ± 0.72	1.08 ± 0.11	26.88 ± 3.95	32.08 ± 3.39
Tac	34.78 ± 2.96	7.83 ± 0.69^**^	1.56 ± 0.13^**^	77.65 ± 5.29^**^	48.44 ± 5.02^*^

Values are presented as the means±SD (*n* = 9 animals/group). FBG, Fasting blood glucose. Crea, Creatinine. ALT, Alanine asminotransferase. ∗*p* < 0.05 and ∗∗*p* < 0.01 compared to Tac and Control group

### GC-MS total ion chromatograms (TICs) of serum and major tissue samples

**Fig. 1:**
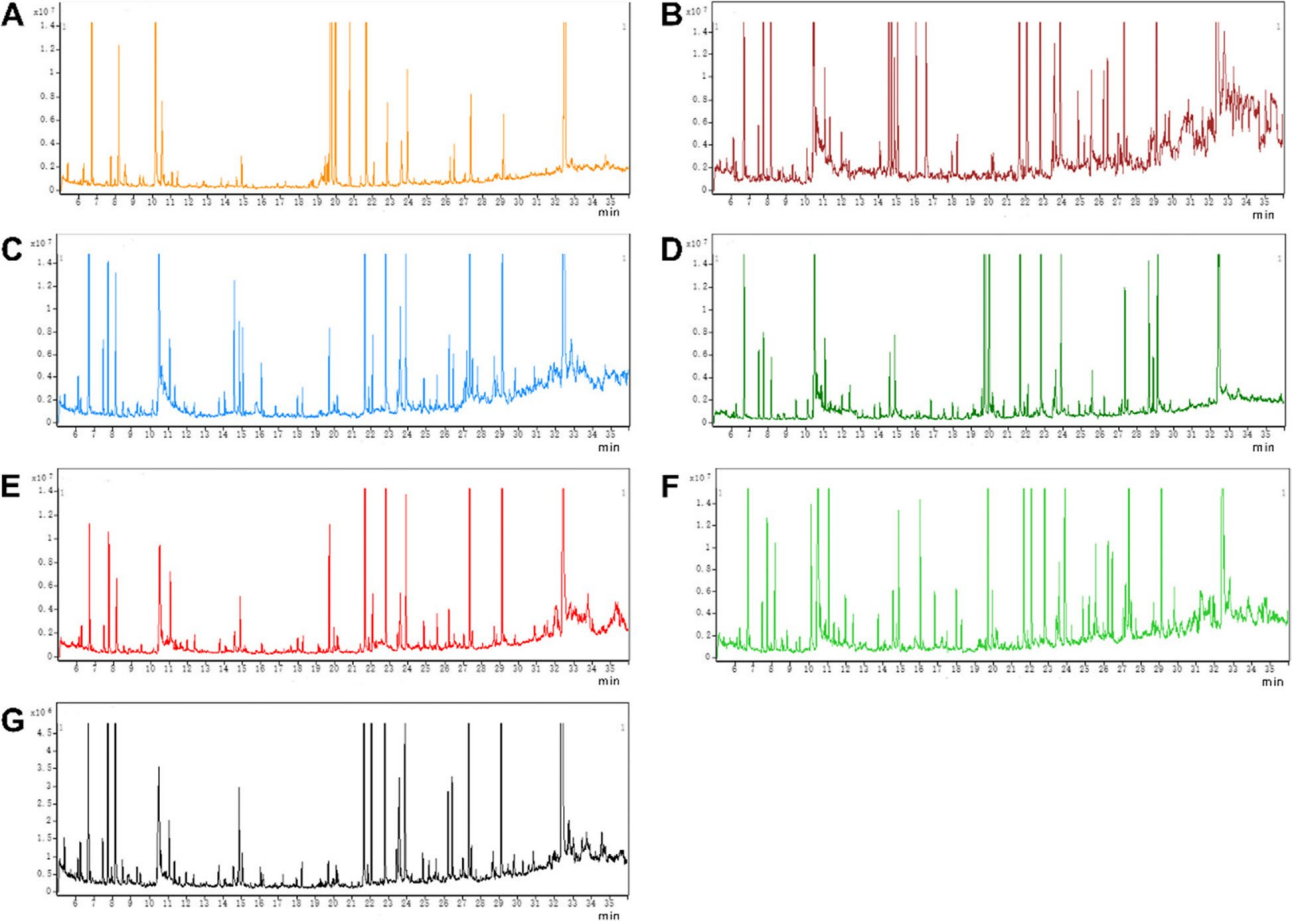
Representative GC − MS total ion chromatograms (TICs) of the serum (**A**), brain tissue (**B**), heart tissue (**C**), liver tissue (**D**), lung tissue (**E**), kidney tissue (**F**), and intestine tissue (**G**) samples from a mixture of the control and Tac-treated mice

Differences in TICs among different samples (serum, brain, heart, liver, lung, kidney and intestines) could be observed in Fig. 1. Typical QC serum and major tissue sample TICs from Tac and control mice revealed strong signals and satisfactory RT reproducibility.

### Multivariate analysis of metabolomic data

**Fig. 2:**
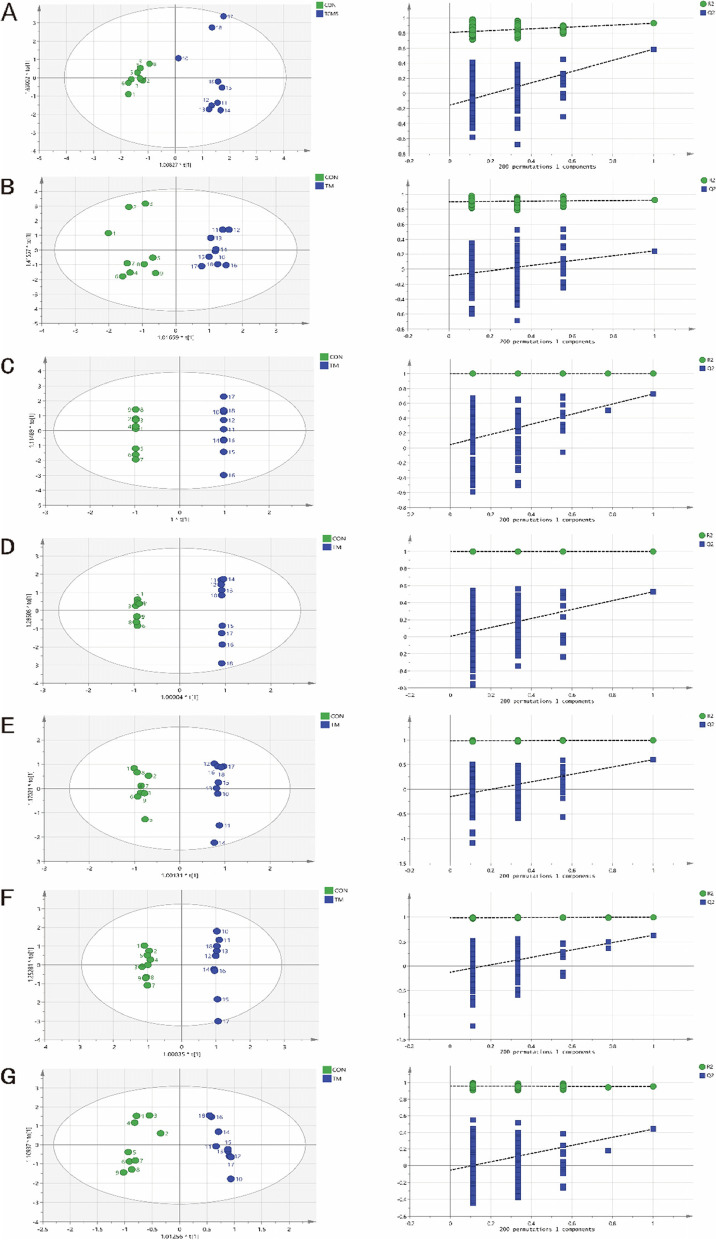
OPLS-DA scores and 200 permutation tests chart for tissue models: serum (**A**), brain tissue (**B**), heart tissue (**C**), liver tissue (**D**), lung tissue (**E**), kidney tissue (**F**), and intestine tissue (**G**)

The OPLS-DA parameters showed effective modeling that distinctively separated the Tac from the control mice(Table 2), parameter values close to 1.0 denoted a stable model with satisfactory predictive ability. Permutation tests were used to further confirm our OPLS-DA models. The Q2-point blue regression line intersected with the vertical axis (on the left) at, or below zero (Fig. 2), indicating the validity of the original model. The OPLS-DA models showed an apparent difference between the Tac group and control group.

**Table 2:** The OPLS-DA parameters in the target main tissues

Tissue	R2X(cum)	R2Y(cum)	Q2(cum)
serum	0.384	0.93	0.587
brain	0.319	0.923	0.246
heart	0.554	1	0.728
liver	0.517	1	0.528
lung	0.307	0.992	0.6
kidney	0.369	0.997	0.632
intestines	0.25	0.952	0.439

The scores of the model parameters, R2X, R2Y, Q2 were close to 1.0

Moreover, OPLS-DA with VIP > 1.0 and *p*-value of t-tests (*p* < 0.05), relative to controls, showed metabolite variations within the Tac mice. In addition, FC (fold change) > 1 indicated that the metabolites had an upward trend, while FC < 1 indicated a downward trend. Table 3 summarizes in detail the data on altered metabolites in major murine tissues, in response to Tac exposure. There were 8 serum metabolites, namely, D-proline, glycerol, D-fructose, D-glucitol, sulfurous acid, 1-monopalmitin (MG (16:0/0:0/0:0)), glycerol monostearate (MG (0:0/18:0/0:0)), and cholesterol. The altered brain metabolites were as follows: butanamide, tartronic acid, aminomalonic acid, scyllo-inositol, dihydromorphine, myo-inositol, and 11-octadecenoic acid. The altered heart metabolites were: acetone and D-fructose. Tac-mediated alterations in the liver included D-glucitol, L-sorbose, palmitic acid, myo-inositol, and uridine. The lung altered metabolites were L-lactic acid, L-5-oxoproline, L-threonine, phosphoric acid, phosphorylethanolamine, D-allose, and cholesterol. The altered metabolites in the kidney were: L-valine and D-glucose.

**Table 3:** List of changed metabolites in the serum, brain, heart, liver, lung and kidney

Tissue	Metabolites	HMDB	VIP	FC
serum	D-Proline	HMDB0003411	1.21	1.87
	Glycerol	HMDB0000131	1.23	1.84
	D-Fructose	HMDB0000660	1.32	1.91
	D-Glucitol	HMDB0000247	1.49	2.37
	Sulfurous acid	HMDB0034829	1.35	2.33
	1-Monopalmitin, MG(16:0/0:0/0:0)	HMDB0011564	1.47	2.37
	Glycerol monostearate, MG(0:0/18:0/0:0)	HMDB0011535	1.18	1.74
	Cholesterol	HMDB0000067	1.24	2.07
brain	Butanamide	HMDB0033870	1.47	0.33
	Tartronic acid	HMDB0035227	1.52	0.44
	Aminomalonic acid	HMDB0001147	1.46	2.01
	Scyllo-Inositol	HMDB0006088	1.46	0.47
	Dihydromorphine	HMDB0060548	1.68	0.31
	myo-Inositol	HMDB0000211	1.54	1.48
	11-Octadecenoic acid	HMDB0003231	1.05	1.19
heart	Acetone	HMDB0001659	2.59	5.09
	D-Fructose	HMDB0000660	2.42	6.65
liver	D-Glucitol	HMDB0000247	1.87	2.49
	L-Sorbose	HMDB0001266	1.67	2.10
	Palmitic Acid	HMDB0000220	1.06	1.34
	myo-Inositol	HMDB0000211	1.46	1.72
	Uridine	HMDB0000296	1.41	1.67
lung	L-Lactic acid	HMDB0000190	1.26	1.52
	L-5-Oxoproline	HMDB0000267	1.60	1.70
	L-Threonine	HMDB0000167	1.30	1.48
	Phosphoric acid	HMDB0002142	1.63	1.93
	Phosphorylethanolamine	HMDB0000224	1.42	1.73
	D-Allose	HMDB0001151	1.72	1.93
	Cholesterol	HMDB0000067	1.59	1.59
kidney	L-Valine	HMDB0000883	1.01	1.35
	D-Glucose	HMDB0000122	2.09	5.04

HMDB, Human Metabolome Database. VIP, variable influence on projection. Fold change, the VCM group/the control group

**Fig. 3:**
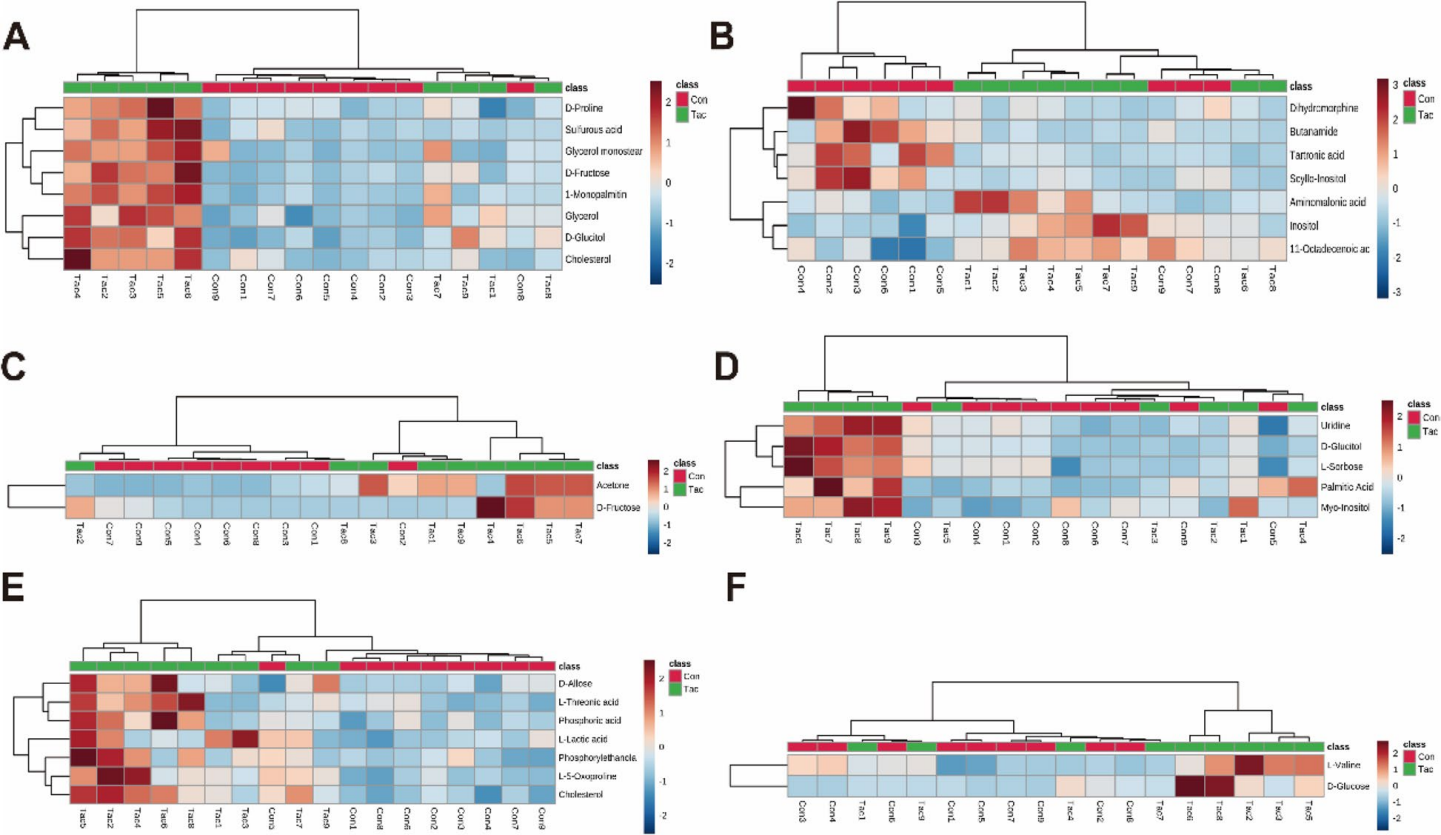
Heatmap of differentially expressed metabolites in Tac and control groups: serum (**A**); brain (**B**); heart (**C**); liver (**D**); lung (**E**); kidney (**F**). Red and blue represent upregulation and downregulation, respectively; the shade is proportional to the degree of change. Rows and columns correspond to samples and metabolites, respectively

The authors also assessed the altered metabolites data via heatmaps (MetaboAnalyst v5.0). A majority of the metabolites were distinctively grouped into two discrete clusters, with minimal overlap (Fig. 3).

### Analysis of metabolic pathways

To further assess the metabolic pathways following Tac exposure, the authors evaluated the metabolites using MetaboAnalyst v5.0 and KEGG database. Metabolic pathways with Raw *P* < 0.5 and Impact > 0 were considered as potential disturbed pathways. The authors observed a singular sulfur metabolism in the serum.

## Discussion

Our study showed significant changes in FBG, Crea, urea and ALT in Tac group (Table 1), suggesting that the Tac displayed a drug toxicity in the major target organs. Herein, the authors examined the metabolic profile of major organs (serum, brain, heart, liver, lung, kidney, and intestines) after Tac exposure. The authors observed alterations in 8, 7, 2, 5, 7, and 2 metabolites in the serum, brain, heart, liver, lung and kidney, respectively, between the Tac and control mice. Further examination of the significance of these metabolites in biological processes can supply additional intel into the Tac toxicity pathophysiology. Based on our analysis, these altered metabolites were the primary mediators of lipid, amino acid, and carbohydrate metabolites. Moreover, even though Tac is not known to produce serious adverse effects on the lipid profile, our data revealed that the lipid metabolism was strongly dysregulated in Tac mice. In fact, cholesterol, glycerol, MG (16:0/0:0/0:0), MG (0:0/18:0/0:0) were markedly altered. Interestingly, unlike our results, a prior study revealed that cholesterol and triglyceride were strongly diminished by Tac exposure [16]. Hence, additional investigations are warranted to elucidate the true effect of Tac exposure on the lipid profile. In addition, the authors demonstrated that the MG (16:0/0:0/0:0) and MG (0:0/18:0/0:0) were elevated, which was likely due to enhanced lipolysis, in response to glucose metabolic disorders. Our data corroborates with the reported dyslipidemic effects of toxic dosages of Tac exposure.

### Gastrointestinal disturbances

Patients with diarrheal illness may demonstrate variability and potential toxicity following Tac exposure [17]. However, in our study, the authors observed no significant metabolite alterations within the intestine. Tac metabolism is regulated by the CYP3A enzyme system, and occurs mostly in the small intestine, liver, and kidney [18]. The upper small intestine is the primary site for CYP3A4-based first-pass metabolism in humans, which may explain the metabolic alternation in the intestine. However, additional studies are warranted to validate our results.

### Post transplanted diabetes mellitus (PTDM)

Post transplanted diabetes is among the most severe Tac-mediated adverse effects [19]. It is brought on by β-cell apoptosis, diminished insulin gene expression, and direct toxicity to the islets of Langerhans [20]. Tac-mediated islet toxicity is most frequent during solid organ transplantation, and it has a prevalence of newly developed diabetes following transplant of up to 30% within the first year [21–23]. Our study uncovered several carbohydrate metabolite alterations in multiple tissues, namely, D-fructose, D-glucitol in the serum, D-Fructose in the heart, D-glucitol in the liver, and D-glucose in the kidney. These alterations indicate carbohydrate metabolism dysfunction following Tac-induced toxicity. Hence, this study confirmed that Tac exposure increases risk of developing PTDM.

### Cardiotoxicity-related metabolic alterations

Although cardiotoxicity is relatively rare with Tac exposure, there are reports of arrhythmia and cardiomyopathy following Tac exposure [24, 25]. Although arteritis of cardiac arteries, hypertension, renal vasoconstriction [26] and reduced nitric oxide formation [27] are potential mechanisms of Tac-mediated toxicity, the true underlying mechanism remains undetermined. In this study, no specific metabolite alterations were observed in the heart tissue, although a marked increase in acetone may be suggestive of Tac-induced toxicity. The heart requires massive energy, and a constant supply of glucose, lipids, and amino acids to generate ATP to sustain a healthy heart beat [15]. Markedly elevated concentrations of ketone bodies like acetone points to the impairment of the TCA cycle, and a switch from glucose oxidation to β oxidation of fatty acids indicates dysregulation within the energy metabolism [28].

### Hepatic lesion-based metabolic alterations

Liver is essential for glycogen storage, protein synthesis, and detoxification [29]. Emerging evidences suggest that Tac exposure elicits liver damage, and the hepatocytes form a ground-glass appearance, which is an early indicator of Tac toxicity [30]. In terms of the biochemical indexes, ALT, alkaline phosphatase, and total bilirubin are established markers of hepatic lesion. Tac is reported to accelerate cholestasis by suppressing biliary excretion of glutathione [31].

Herein, the authors demonstrated marked elevation of L-sorbose, palmitic acid, myo-inositol, and uridine in the hepatic tissue. Among these metabolites, palmitic acid concentration was previously reported to be elevated during hepatotoxicity in drug toxicity-based investigations [32, 33]. This increase indicates a rise in de novo synthesis of fatty acids via an alternate axis involving ß-oxidation [34, 35]. Moreover, being a FFA, palmitic acid elicits an elevated hepatic cytotoxic outcome [36]. Uridine minimizes cytotoxicity and improves neurophysiological activities [37, 38]. However, recently, clinical data suggested a direct association between plasma uridine levels and insulin resistance in humans [39, 40]. Prior investigations reported that short uridine exposure accelerated hepatic insulin resistance in C57BL/6 J mice [41]. Combined with PTDM of Tac, uridine dysregulates glucose metabolism in the liver.

### Lung toxicity-related metabolic changes

Currently, there are limited reports on Tac-mediated pulmonary toxicity. Akhtar reported that Tac causes structural distortion of the lungs in rats, characterized by alveolar cells necrosis, bronchiolar wall thickening, and interstitial round cell infiltration, which confirms the toxic potential of Tac in lungs [42]. Additionally, a prior investigation revealed that the oxidative stress parameters and proinflammatory markers were significantly increased in rats with Tac-treatment, compared to controls [43].

In this study, the authors presented the metabolite alterations following Tac toxicity in lung tissues. Our study assessed the metabolic alterations of Tac-induced lung toxicity, and showed marked elevations in the levels of L-lactic acid, L-5-oxoproline, L-threonine, and phosphorylethanolamine in the Tac mice, relative to controls. Lactic acid, produced via aerobic glycolysis, is a potential early indicator of a reversible state in critically ill patients [44]. L-5-oxoproline is an endogenously formed metabolite that elicits adverse effects at chronically elevated dosages. When present at high levels, L-5-oxoproline and lactic acid serve as metabotoxins, which may present as a toxicity index for Tac. Threonine is an immunostimulant which positively regulates thymus gland development, as well as cell immune defense activity. However, the role of threonine in Tac-induced lung toxicity remains unclear.

### Neurotoxicity-related metabolic alterations

Tac-related neurotoxicity, mediated by elevated blood concentrations of Tac, produces headache, tremor, delirium, and peripheral neuropathy [45]. In a large prospective study, significant neurological undesirable events were correlated with high plasma levels in > 50% patients [46]. Metabolic investigations involving the whole brain of Tac-exposed mice are relatively rare. Hence, more research is warranted in this area to supplement what is known about Tac-mediated toxicity and its related mechanisms in major organs.

Herein, Tac-exposed mice showed alterations in aminomalonic acid, scyllo-inositol, dihydromorphine, myo-inositol, and 11-octadecenoic acid. Aminomalonic acid strongly inhibits L-asparagine synthase activity, and is up-regulated in the urine of anxiety and major depressive disorders-diagnosed individuals [47]. Multiple metabolomic investigations also revealed that altered serum aminomalonic acid concentration was closely correlated with neuropsychiatric disorders, ketamine overdose, and aortic aneurysm [48], thus forming a link between aminomalonic acid and various diseases and toxicities. This study revealed that aminomalonic acid can serve as a potential indicator of Tac-mediated neurotoxicity. Scyllo-inositol improves brain cognitive function, reverses memory deficits, and minimizes amyloid-beta (Aβ) plaque within brains of mice [49]. Thus, the low levels of scyllo-inositol in our study may be another indicator of Tac-mediated neurotoxicity.

### Nephrotoxicity-related metabolic alterations

Nephrotoxicity is the most common and clinically significant adverse Tac reaction, and it occurs in almost half of the Tac-treated patients [18]. Multiple reports demonstrated glycosuria to be an effective bioindicator of acute renal toxicity [50, 51]. Herein, D-Glucose was observed to be significantly altered, suggesting that the nephrotoxicity was successfully induced by Tac at the selected concentration. Additionally, the authors also observed marked increases in L-valine in our Tac-treated mice. Alteration in L-valine was also found in other toxic kidney’s injury studies [52, 53]. L-valine is a branched-chain amino acid that links only to carbohydrates (glycogenic) [54]. Valine is intricately linked to insulin resistance, and elevated serum valine concentrations were reported in both diabetic mice and humans [55, 56]. Thus, it is obvious that L-valine participates in the progression of Tac-induced nephrotoxicity.

Herein, the authors systematically assessed Tac-toxicity using GC − MS-based profiling of target tissues. Toxicity is the most common and significant result of elevated blood Tac concentrations. Owing to its narrow therapeutic window, close monitoring of this therapeutic drug is imperative for treatment individualization. Unfortunately, it is still difficult to predict a particular dose that is optimal for a specific patient. Therefore, the effect of different dosages on metabolic profiles needs further investigation.

In conclusion, in this study, the authors reported an extensive metabolic profile following Tac exposure in mice. The authors demonstrated that the altered metabolism involved cellular processes like lipid, amino acid, and carbohydrate metabolism, which may, in turn, provide certain insight into the pathogenesis of Tac-mediated toxicity. Our work will greatly benefit clinicians and researchers, particularly in guiding Tac dosing, and to further understand the toxicological mechanism of Tac.

## Data Availability

The datasets used and analyzed during the current study are available from the corresponding author on reasonable request.

## Abbreviations

Aβ: Amyloid-beta
ALT: Alanine aminotransferase
BSTFA: N,O-bis- (trimethylsilyl)trifluoroacetamide
Crea: creatinine
FBG: Fasting blood glucose
FC: Fold change
GC-MS: Gas chromatography−mass spectrometry
IS: Internal standard
KEGG: Kyoto Encyclopedia of Genes and Genomes
KM: Kunming
MG: Glycerol monostearate
OPLS: Orthogonal projections to the latent structure
PTDM: Post transplanted Diabetes Mellitus
QC: Quality control
RT: Retention time
Tac: Tacrolimus
TCA cycle: Tricarboxylic acid cycle
TICs: Total ion chromatograms
TMCS: Trimethylchlorosilane
VIP: Variable importance in projection.
